# Scalable clinical proteomics from DNA/RNA extraction flowthroughs enables integrated proteogenomic profiling from a single cancer biopsy

**DOI:** 10.1186/s12014-026-09632-1

**Published:** 2026-09-20

**Authors:** Filip Mundt, Annelaura Bach Nielsen, Juanjuan Wang, Josephine Kerzel Duel, Christina Westmose Yde, Martina Amnitzbøll Eriksen, Ulrik Lassen, Finn Cilius Nielsen, Kristoffer Rohrberg, Matthias Mann

**Affiliations:** 1 Proteomics Program, Novo Nordisk Foundation Center for Protein Research, University of Copenhagen, Faculty of Health and Medical Sciences, Copenhagen, Denmark; 2 Department of Clinical Biochemistry Copenhagen University Hospital, Bispebjerg and Frederiksberg Hospital, Copenhagen, Denmark; 3 Rigshospitalet, Copenhagen University Hospital , Denmark; 4 Department of Proteomics and Signal Transduction, Max Planck Institute of Biochemistry, Martinsried, Germany

## Abstract

**Background:**

Precision oncology has largely relied on genomic and transcriptomic sequencing to guide therapy selection in patients with advanced cancer. However, these molecular layers do not fully capture the functional state of tumors, as proteins largely represent the active effectors of cellular processes and signaling pathways. Integrating proteomic and phosphoproteomic measurements with genomic data has therefore emerged as an important objective in translational oncology. In routine clinical workflows, tumor biopsies are frequently processed using DNA/RNA extraction kits such as the Qiagen AllPrep system, which generates a protein-rich flowthrough that is typically discarded.

**Methods:**

Here we systematically evaluated whether the residual protein fraction generated during Qiagen AllPrep DNA/RNA extraction can serve as a reliable source for mass spectrometry–based proteomics in clinical tumor biopsies. Within the Copenhagen Prospective Personalized Oncology (CoPPO) precision oncology trial, residual protein fractions from metastatic cancer biopsies were recovered, precipitated, and processed using optimized workflows for liquid chromatography–mass spectrometry. We established procedures for protein recovery, digestion, and peptide cleanup, and evaluated multiple acquisition strategies including data-dependent acquisition (DDA) and data-independent acquisition (DIA). We further assessed sample stability during long-term storage, analytical reproducibility, scalability, and compatibility with phosphoproteomics.

**Results:**

Optimized single-shot workflows consistently generated deep proteomes from AllPrep-derived biopsy material, quantifying more than 10,000 protein groups in DIA analyses. Protein integrity was preserved in samples stored at − 80 °C for up to five years. The resulting proteomes retained tissue-associated biological signatures and quantified protein products corresponding to approximately 71% of genes represented on the FoundationOne^®^ CDx panel. Phosphoproteomic analyses identified more than 10,000 phosphorylation sites associated with key oncogenic signaling pathways including AKT1, BRAF, and EGFR. Implementation of short liquid chromatography gradients enabled high-throughput analysis of up to 60 proteomes per day without compromising data quality.

**Conclusions:**

Residual protein fractions from Qiagen AllPrep DNA/RNA extraction provide a scalable resource for deep proteomic and phosphoproteomic profiling from the same biopsy used for genomic and transcriptomic analyses. This workflow enables integrated proteogenomic characterization from a single clinical sample and provides a framework for integrated proteogenomic studies in precision oncology.

**Supplementary Information:**

The online version contains supplementary material available at https://doi.org/10.1186/s12014-026-09632-1.

## Introduction

Cancer arises through the accumulation of genomic alterations that disrupt cellular regulatory networks and promote uncontrolled growth. Over the past decade, large-scale genomic and transcriptomic sequencing efforts have greatly expanded our understanding of tumor biology and have enabled the development of precision oncology strategies in which molecular alterations are matched to targeted therapies. These approaches are increasingly implemented in clinical practice, particularly for patients with metastatic disease, where molecular profiling may identify treatment opportunities beyond standard care [1, 2].

Despite these advances, the clinical benefits of genomic precision oncology have often been limited. One explanation is that genomic and transcriptomic measurements do not fully reflect the functional state of cancer cells. Proteins are the direct executors of cellular processes, and their abundance, activity, and post-translational modification states ultimately determine signaling dynamics and therapeutic responses. Consequently, integrating proteomic measurements with genomic and transcriptomic data may provide a more comprehensive molecular characterization of tumors [3].

Mass spectrometry (MS)–based proteomics has undergone substantial technological development in recent years, enabling increasingly sensitive, robust, and high-throughput analysis of complex biological samples. Modern workflows now allow deep and reproducible quantification of thousands of proteins from clinical material, making proteomics increasingly compatible with translational research and clinical investigations [4–11]. These advances have stimulated growing interest in clinical proteogenomics, in which genomic and proteomic measurements are combined to characterize tumor biology and therapeutic vulnerabilities.

Most clinical proteomic analyses currently rely on formalin-fixed paraffin-embedded (FFPE) tissue samples, which are routinely generated for histopathological evaluation and have become amenable to MS-based analysis through optimized workflows [12, 13]. However, a common challenge in multi-omics studies is that proteomic and genomic measurements are often performed on separate biopsy fragments. Because tumors can exhibit substantial spatial heterogeneity, such separation can complicate the interpretation of correlations between molecular layers [14, 15].

At the Phase I Unit at Rigshospitalet in Copenhagen, Denmark, patients with advanced metastatic cancer and no remaining standard treatment options are enrolled in the Copenhagen Prospective Personalized Oncology (CoPPO) clinical trial [2]. In this program, metastatic tumor biopsies undergo extensive genomic and transcriptomic profiling, and the resulting data are integrated into clinical reports that inform treatment decisions in national Molecular Tumor Boards. DNA and RNA extraction is routinely performed using the Qiagen AllPrep kit, which allows sequential isolation of nucleic acids from the same sample. During this process, a protein-rich flowthrough is generated as a by-product of the extraction procedure.

Although this protein fraction is typically discarded, it represents a potentially valuable source of material for proteomic analysis. Recognizing this opportunity, the Phase I Unit has prospectively preserved these protein-rich flowthrough fractions at − 80 °C for more than a decade, creating a unique biobank of clinical tumor samples suitable for retrospective proteomic investigation.

In the present study, we systematically evaluated the feasibility of performing deep proteomic and phosphoproteomic analyses from these residual AllPrep protein fractions. We established optimized workflows for protein recovery, digestion, and peptide purification, assessed the impact of long-term storage on protein integrity, and evaluated different mass spectrometry acquisition strategies to maximize proteome depth and reproducibility. Furthermore, we investigated the scalability of the workflow and its compatibility with high-throughput clinical proteomics platforms.

Our results demonstrate that the protein-rich flowthrough generated during routine DNA/RNA extraction can serve as a source for deep proteomic analysis from clinical tumor biopsies. Importantly, this approach enables proteomic, phosphoproteomic, genomic, and transcriptomic measurements to be obtained from the same biopsy specimen, providing a practical framework for integrated proteogenomic profiling in precision oncology.

## Results

### Recovery of proteomic information from AllPrep protein fractions

Tumor biopsies processed with the Qiagen AllPrep DNA/RNA extraction kit are lysed using guanidine-HCl–based buffers that efficiently disrupt cellular structures and inhibit proteolytic degradation. During the sequential purification of DNA and RNA, a protein-containing flowthrough is generated that can be recovered and precipitated using the Advanced Protein Purification (APP) buffer supplied with the kit.

**Fig. 1:**
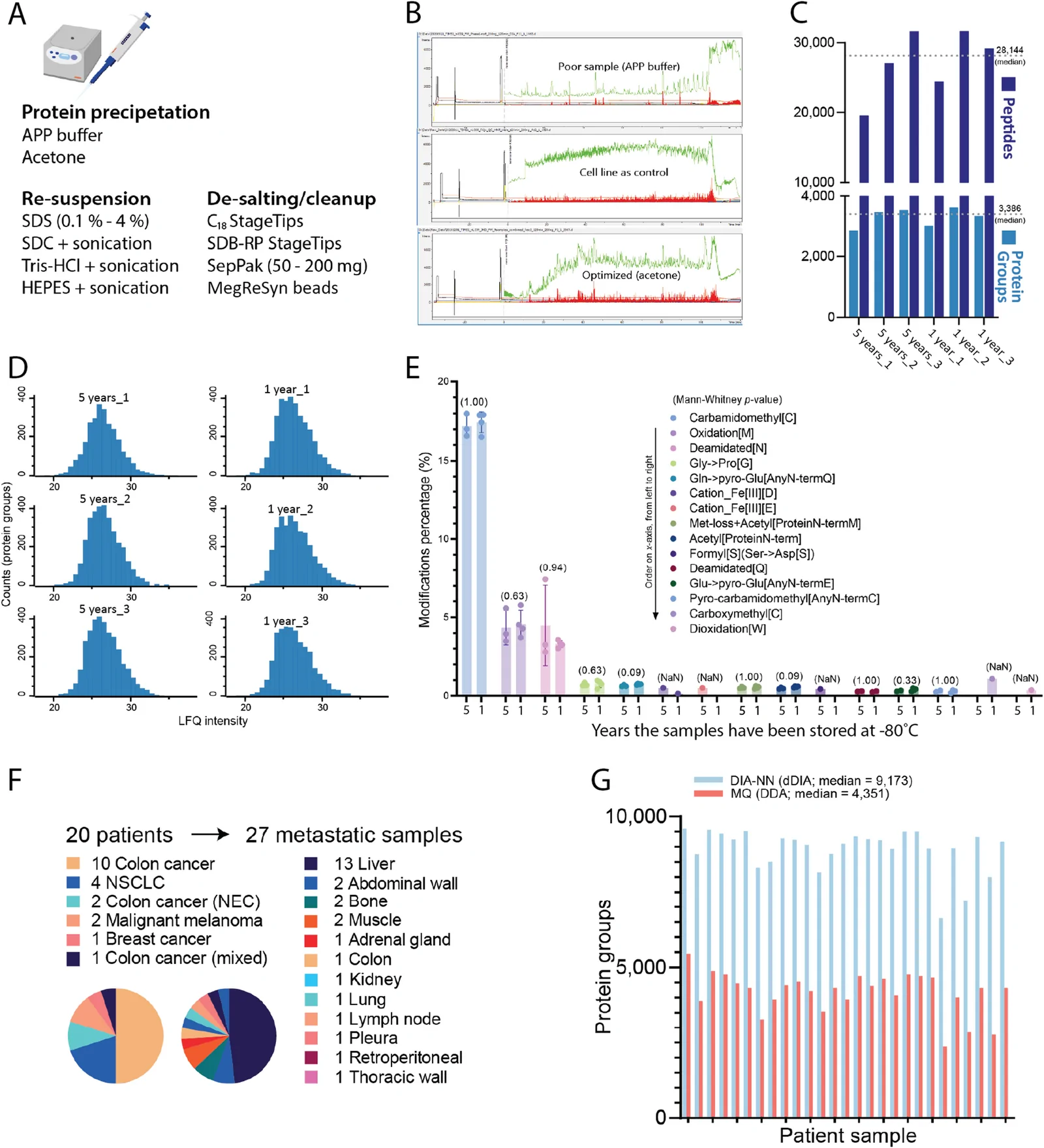
Optimization of workflows for proteomic analysis of AllPrep-derived protein fractions. (**A**) Workflows evaluated for generating MS-compatible peptides from protein-rich flowthrough fractions obtained during Qiagen AllPrep DNA/RNA extraction of tumor biopsies, including alternative solubilization, digestion, and cleanup strategies. (**B**) Representative chromatograms comparing peptide recovery using APP precipitation, a HeLa quality-control sample, and acetone precipitation. (**C**) Identified peptides and protein groups from six tumor-derived fractions following optimized acetone precipitation and digestion after varying storage durations at −80 °C. Dark blue bars represent peptide counts, while light blue bars are protein groups. (**D**) Log₂-transformed protein abundance distributions showing comparable proteome profiles across the six samples. (**E**) Post-translational modification frequencies identified by open modification search (pFind). No significant differences were observed between samples stored for one or five years at −80 °C (Student’s *t*-test), supporting protein integrity during long-term storage. (**F**) Workflow for analysis of 27 AllPrep-derived protein fractions from metastatic tumor biopsies of patients with BRAF V600E–mutated cancers, representing multiple tumor types and clinical time points. (**G**) Comparison of DDA and DIA protein identifications. DIA increased proteome coverage and reduced missing values

However, when we initially attempted to process these fractions for proteomic analysis, we observed that direct resuspension of the resulting protein pellets often led to incomplete solubilization and limited peptide recovery. To address this limitation, we systematically evaluated alternative strategies for protein solubilization, digestion, and peptide cleanup (Fig. 1A).

Multiple buffer systems were tested for resuspension, including sodium deoxycholate (SDC), Tris-HCl, and urea-based buffers, combined with mechanical disruption by sonication and enzymatic digestion using Lys-C and trypsin. Peptides were subsequently purified using either SDB-RPS StageTips or Sep-Pak^®^ C18 cartridges [16].

Initial analyses indicated that although protein concentrations measured by NanoDrop spectroscopy ranged between approximately 0.1 and 3.0 µg/µL, the resulting MS spectra were of relatively low quality, yielding identification of only around 500 proteins per sample. Implementation of S-Trap™ digestion workflows improved the results modestly, increasing the number of identified proteins to approximately 1,000, but the incompatibility of SDS with guanidine-based lysis buffers limited the overall performance of this approach.

Subsequent optimization demonstrated that acetone precipitation followed by resuspension in SDC or urea buffers and peptide purification using Sep-Pak cartridges yielded substantially improved results. Under these conditions, peptide chromatograms exhibited markedly enhanced signal intensity and complexity (Fig. 1B), indicating efficient protein recovery and digestion.

Typically, approximately 800 µL of flowthrough was obtained from each biopsy. Processing of 250 µL of this material yielded roughly 50 µg of total protein, which proved sufficient for repeated proteomic analyses.

To evaluate the performance of this workflow, six patient samples representing diverse cancer types and storage durations were analyzed using data-dependent acquisition on an Orbitrap Exploris 480 instrument. Median quantification reached approximately 3,300 protein groups and 28,000 peptides per sample (Fig. 1C), with highly comparable protein abundance distributions across all samples (Fig. 1D).

This initial single-shot DDA experiment was designed to establish the feasibility and reproducibility of recovering proteomic information from the AllPrep fractions rather than to maximize proteome depth. Analytical depth was subsequently evaluated using DIA-based acquisition strategies, as described below.

Open modification searches further confirmed that the most frequent post-translational modifications were carbamidomethylation of cysteine, methionine oxidation, and N-terminal deamidation (Fig. 1E). Importantly, no increase in oxidative damage or other degradation signatures was observed in samples stored for up to five years at − 80 °C, indicating that protein integrity is preserved during long-term storage. For later high-throughput experiments, the recovered AllPrep protein fractions were also adapted to automated bead-based protein aggregation capture using MagReSyn Hydroxyl beads on a KingFisher Flex platform, as described below.

We next evaluated how DIA-based acquisition affected analytical depth and completeness.

To evaluate the analytical performance of DIA-based acquisition, we analyzed a larger cohort of 27 protein fractions derived from BRAF V600E–mutated tumors representing multiple cancer types, biopsy sites, and clinical time points (Fig. 1F). These samples were collected over a 5-year period and the numbers of quantified peptides and protein groups were similar across all years, confirming that long-term storage did not adversely affect data quality. Clustering of the samples was driven primarily by colon cancers and liver metastases (Supplementary Fig. 1A-B).

To improve completeness [17, 18], we employed data-independent acquisition (DIA) instead of DDA, using a timsTOF Pro mass spectrometer with PASEF technology [19–21]. While DDA analysis (ddaPASEF, MaxQuant [22]) quantified a median of 4,351 protein groups per sample, the DIA workflow (diaPASEF, DIA-NN [23]) substantially increased coverage to a median of 9,173 protein groups (Fig. 1G). In addition to increasing proteome depth, DIA analysis reduced missing values across samples from 42% to approximately 20%.

These results establish that AllPrep protein fractions can provide stable and accessible material for proteomic analysis of archived clinical tumor samples. We next evaluated how analytical throughput affected proteome coverage.

### Short-gradient DIA for increased sample throughput

We next evaluated the trade-off between analytical depth and sample throughput by applying shorter liquid chromatography gradients on the Evosep LC platform [24].

**Fig. 2:**
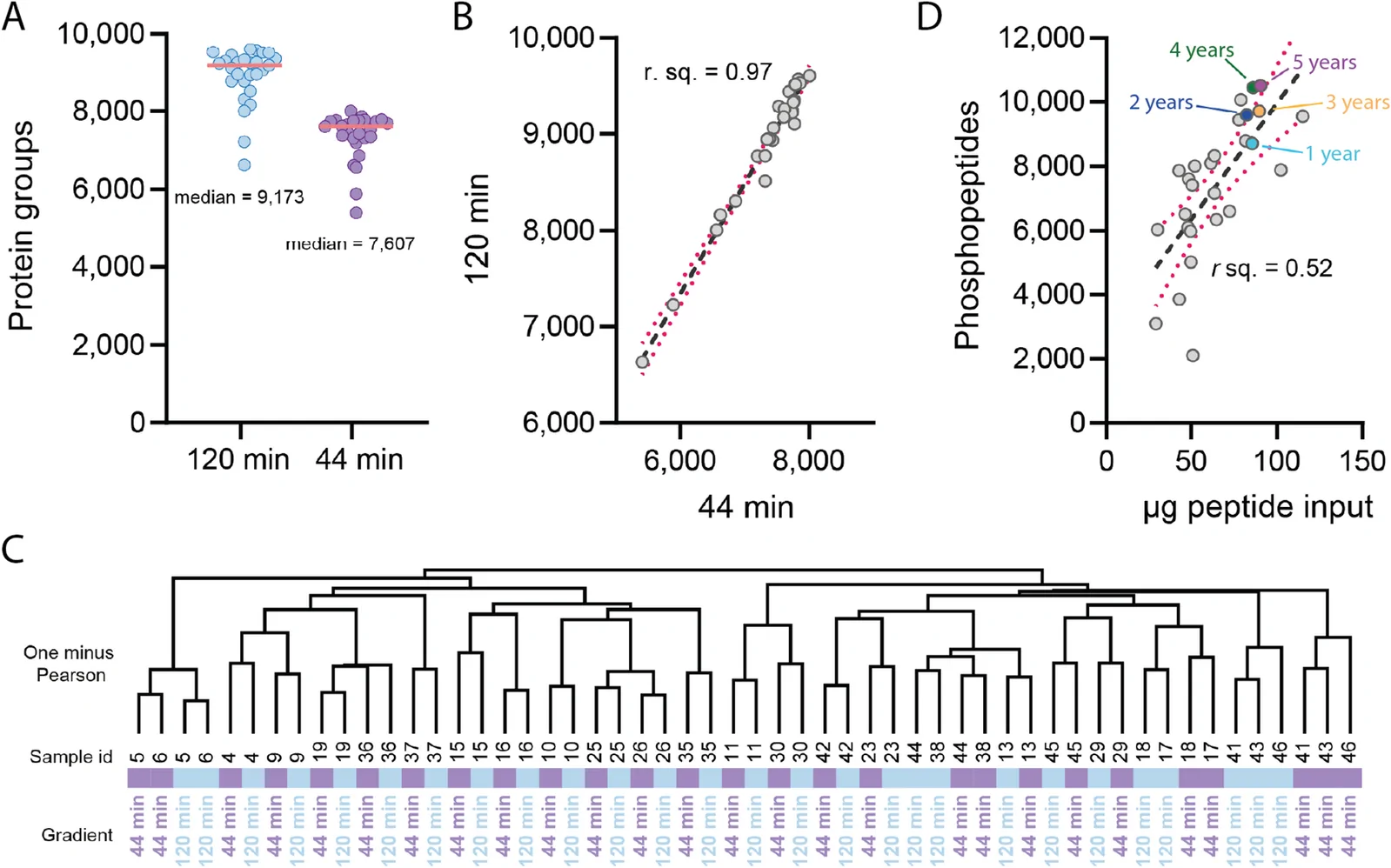
High-throughput proteomic and phosphoproteomic analysis of AllPrep-derived tumor samples. (**A**) Comparison of protein identification numbers obtained using 120-minute and 44-minute liquid chromatography gradients. Shorter gradients increased throughput while maintaining substantial proteome depth. (**B**) Correlation of protein quantification between the two gradient lengths (Pearson r² = 0.97), demonstrating consistent quantitative measurements across acquisition methods. (**C**) Hierarchical clustering of proteomic datasets generated using different gradient lengths, showing preservation of biological sample groupings. (**D**) Phosphoproteomic analysis of enriched peptides demonstrating increasing phosphosite identification with increasing peptide input (29–115 µg). Storage duration did not affect phosphopeptide yield. Black dashed lines indicate linear regression fits and red dotted lines indicate 95% confidence intervals. These experiments demonstrate that AllPrep-derived protein fractions are compatible with scalable clinical proteomics workflows and phosphoproteomic analyses

Using a 44-minute gradient corresponding to approximately 30 samples per day, we analyzed samples using diaPASEF acquisition on a timsTOF SCP instrument. Under these conditions, a median of 7,607 proteins were quantified per sample, representing approximately 82% of the depth achieved using 120-minute gradients (Fig. 2A).

Quantitative measurements obtained using the two gradient lengths were highly correlated (r² = 0.97) (Fig. 2B), indicating that shorter gradients preserved quantitative accuracy. Replicate analyses demonstrated strong reproducibility, with average Pearson correlations of 0.95 ± 0.01 between repeated measurements.

Hierarchical clustering of the resulting datasets produced nearly identical sample groupings for both gradient lengths (Fig. 2C), demonstrating that the shorter acquisition method retained proteomic patterns while substantially increasing sample throughput. These results suggest that the workflow is compatible with high-throughput clinical proteomics applications.

### Phosphoproteomic profiling of AllPrep-derived samples

Because phosphorylation is a central regulatory mechanism in oncogenic signaling pathways [3], we next evaluated whether AllPrep-derived samples could also support phosphoproteomic analyses.

Residual peptides from the BRAF-mutant cohort were subjected to immobilized metal affinity chromatography (IMAC) enrichment using Fe(III)-NTA cartridges on an automated AssayMAP platform. Peptide inputs ranged from 29 µg to 115 µg per sample. Following enrichment, phosphopeptides were analyzed using DIA acquisition on an Orbitrap mass spectrometer.

Across the analyzed samples, between 2,101 and 10,492 phosphopeptides were quantified per sample. Phosphosite identification scaled with peptide input, plateauing at approximately 75–100 µg of starting material (Fig. 2D). This input range represents the approximate plateau observed in this dataset rather than a minimum input requirement; phosphopeptides were successfully quantified from samples with inputs as low as 29 µg, although with reduced depth. Importantly, no systematic differences in phosphopeptide yield were observed between samples stored for different durations, further supporting the stability of the stored protein fractions (Fig. 2D).

Independent clustering of the phosphoproteomic profiles showed biological groupings broadly consistent with those observed in the corresponding proteome analyses (Supplementary Fig. 2). In total, approximately 20,000 phosphorylation sites were mapped to 4,839 genes. Among these, phosphorylation sites were detected on protein products corresponding to 130 genes represented on the FoundationOne^®^ CDx panel. Proteome and phosphoproteome datasets were processed using different analysis pipelines and were therefore considered as complementary molecular layers rather than directly normalized quantitative measurements.

Among these were phosphorylation events in several key regulatory proteins, including AKT1 Ser124, APC Ser2449, BRAF Ser151, Ser365, and Ser729, as well as EGFR Thr693 and Ser1166. These phosphorylation sites have been implicated in oncogenic signaling and therapeutic response [25–38].Together, these results demonstrate that AllPrep-derived material is compatible with phosphoproteomic profiling, while also showing that achievable phosphoproteomic depth depends on available peptide input.

### High-throughput deep proteome coverage using Orbitrap Astral

**Fig. 3:**
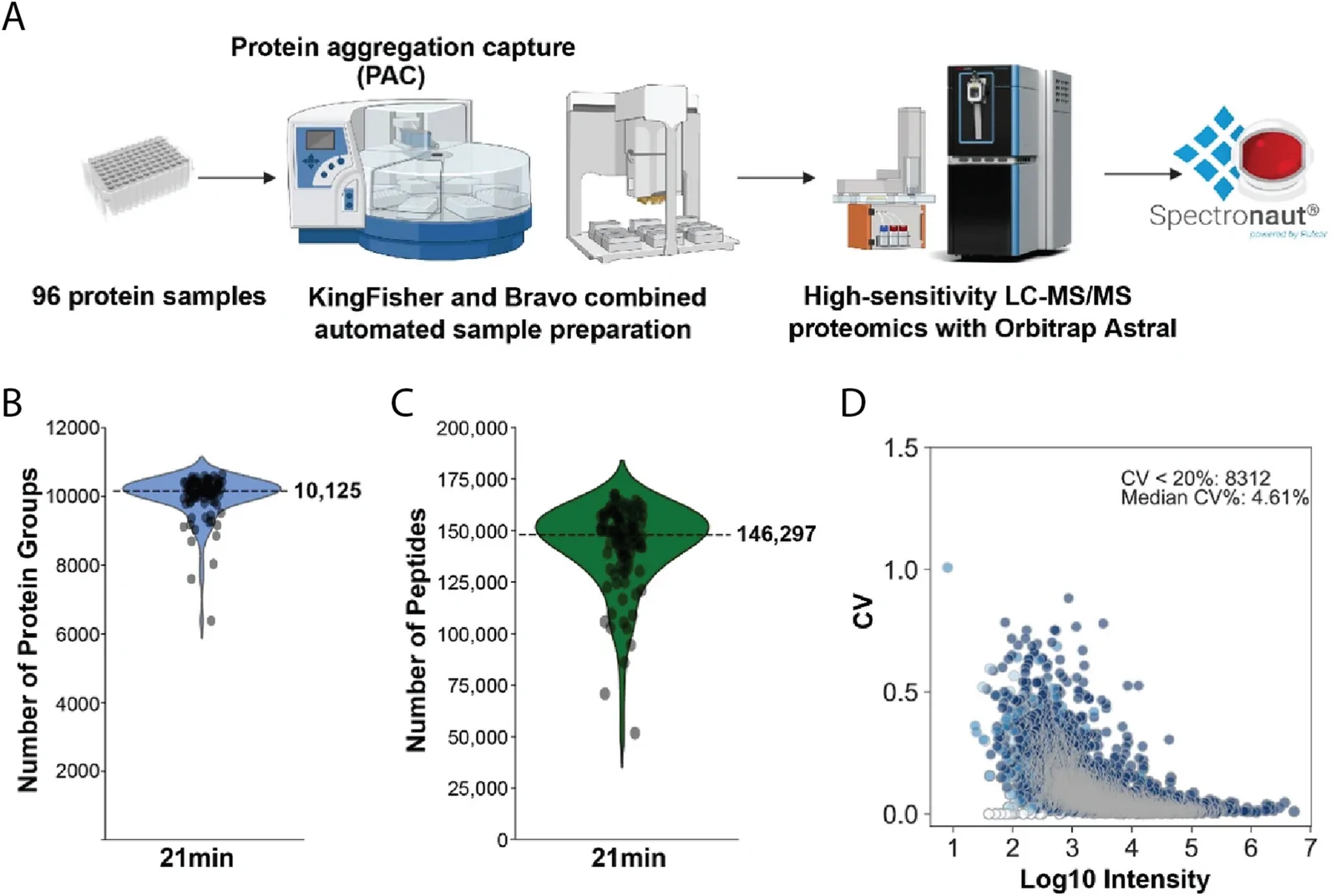
Deep proteome profiling using high-throughput Orbitrap Astral analysis. (**A**) Workflow for automated high-throughput proteomics using Evosep liquid chromatography coupled to the Orbitrap Astral mass spectrometer. Automated 96-well sample preparation was implemented using the KingFisher Flex platform. (**B**) Numbers of quantified protein groups obtained from metastatic tumor biopsies using 21-minute liquid chromatography gradients corresponding to approximately 60 samples per day. (**C**) Numbers of quantified peptides detected in the same analyses, illustrating deep proteome coverage despite short chromatographic gradients. These results demonstrate that AllPrep-derived protein fractions are compatible with next-generation high-throughput proteomics platforms capable of generating deep proteome datasets from large clinical cohorts. (**D**) Distribution of coefficients of variation across quantified proteins, demonstrating high analytical reproducibility

To evaluate the workflow on a current high-throughput proteomics platform, we analyzed 96 AllPrep-derived metastatic tumor samples using an Orbitrap Astral mass spectrometer [39, 40] coupled to the Evosep LC system. For high-throughput Astral analysis, the AllPrep-derived protein fractions were processed using an automated bead-based protein aggregation capture workflow on a KingFisher Flex platform (Fig. 3A).

Peptides were analyzed using a 21-minute liquid chromatography gradient corresponding to approximately 60 samples per day. Despite the short gradient, the workflow achieved a median quantification of 10,197 protein groups per sample (Fig. 3B). Peptide counts reached a median of 125,348 per sample (Fig. 3C), corresponding to an average of 12.3 peptides per quantified protein.

The resulting datasets showed low coefficients of variation (approximately 4.6%), demonstrating strong analytical reproducibility (Fig. 3D). Overall, this configuration achieved a rate of approximately 485 quantified proteins per minute of analysis, illustrating the potential of the workflow for large-scale clinical proteomics studies.

### Biological and clinical information captured in AllPrep-derived proteomes

Having established the analytical feasibility, depth, reproducibility, and scalability of proteomic profiling from AllPrep-derived fractions, we next evaluated whether the resulting proteomes retained biologically and clinically meaningful information.

**Fig. 4:**
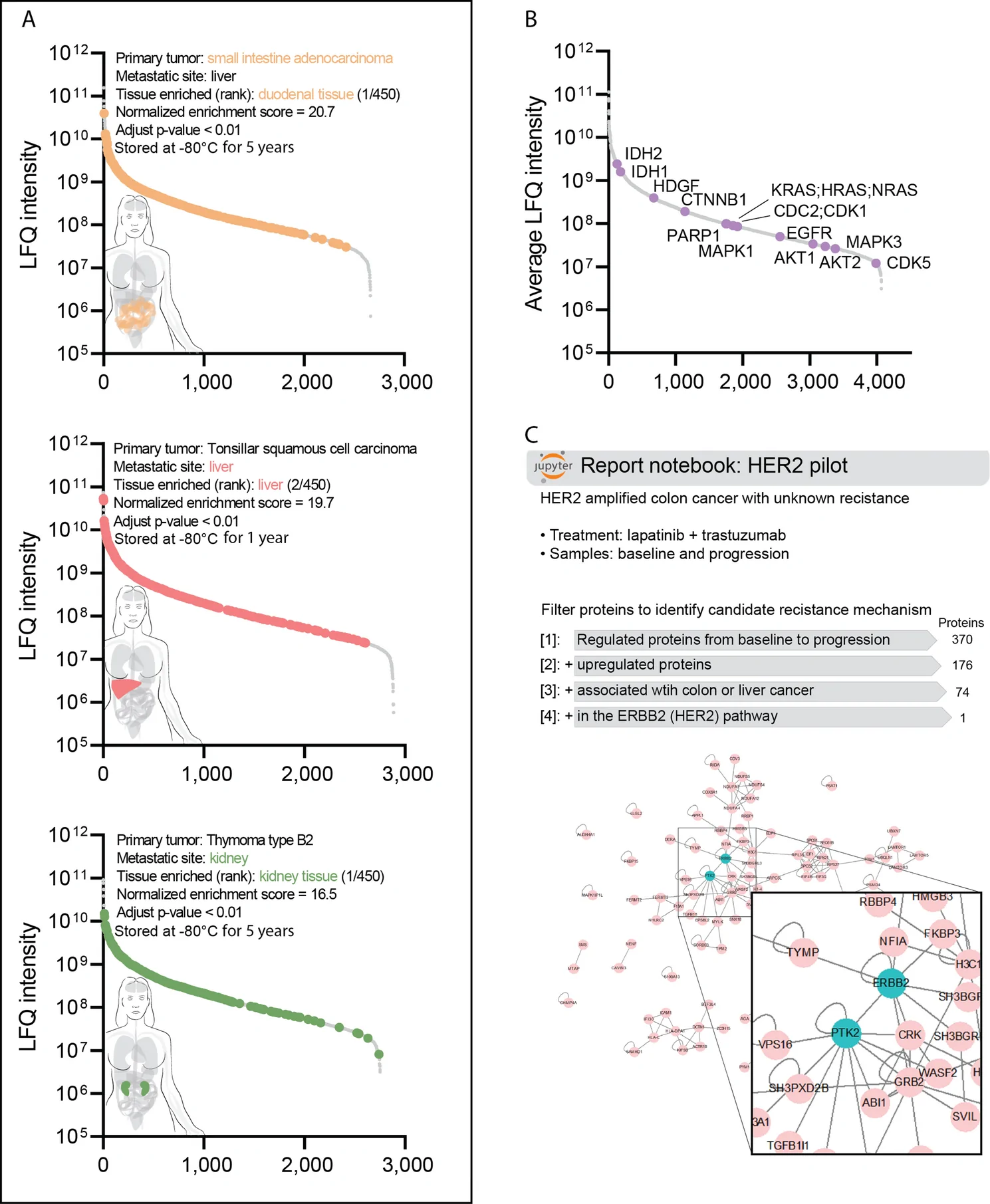
Preservation of biologically informative proteomic signals in AllPrep-derived tumor samples. (**A**) Single-sample gene set enrichment analysis (ssGSEA) using MSigDB C8 cell-type signatures showing enrichment of tissue-associated signatures consistent with the known clinical tumor diagnoses and biopsy sites. LFQ intensities were used as input for enrichment analysis. (**B**) Quantification of selected cancer-associated proteins detected in AllPrep-derived proteomes. Purple dots indicate proteins detected in at least one of the six samples analyzed. (**C**) Example application of the workflow to investigate therapeutic resistance in a patient with HER2-amplified colorectal cancer. A Clinical Knowledge Graph (CKG) network visualization highlights PTK2/FAK1 as a hypothesis-generating candidate associated with the progression proteome. These analyses demonstrate that proteomes derived from AllPrep flowthrough fractions retain biologically meaningful information suitable for downstream clinical proteomics analyses

The six initial samples analyzed represented metastatic tumors originating from several cancer types, including small intestine adenocarcinoma, tonsillar squamous cell carcinoma, and thymoma, obtained from metastatic sites such as liver, lymph node, and kidney. To assess whether AllPrep-derived proteomes retained expected tissue-associated biological signals, we performed single-sample gene set enrichment analysis (ssGSEA) using cell-type signature sets from the MSigDB v7.4 C8 database. Using the known clinical tumor diagnoses and biopsy sites as reference information, enrichment patterns corresponded well with expected tissue-associated signatures (Fig. 4A). For example, intestinal gene signatures were enriched in samples derived from intestinal cancers, while lung-related signatures were enriched in non–small cell lung cancer samples. These results indicate that biologically interpretable tissue-associated signals are retained in the proteomes derived from these stored protein fractions.

The proteomic data also quantified several well-characterized cancer-associated proteins, including EGFR, IDH1/2, AKT1/2, and MAPK1/3 (Fig. 4B). Their detection illustrates the analytical depth of the workflow and its ability to measure proteins of established relevance to cancer biology. Protein detection alone does not establish the presence, pathogenicity, or clinical actionability of corresponding genomic alterations.

To further evaluate the potential clinical relevance of these data, we examined serial biopsies from a patient with ERBB2 (HER2/neu)–amplified colorectal cancer who had progressed during treatment with trastuzumab and lapatinib. Genomic analyses of the progression biopsy did not reveal new resistance-associated mutations. We therefore compared proteomic profiles between baseline and progression samples using single-run DDA analyses.

Across the two samples, more than 3,700 proteins were quantified. We focused on proteins showing the largest abundance increases in the progression biopsy, using the top 10% of increased proteins as candidates for exploratory analysis. Integration with the Clinical Knowledge Graph (CKG) was used to filter proteins associated with colon or liver cancer, HER2 signaling, or resistance to trastuzumab and lapatinib. This analysis highlighted focal adhesion kinase (PTK2/FAK1) as a hypothesis-generating candidate associated with the progression proteome (Fig. 4C).

FAK1 has previously been linked to trastuzumab resistance in ERBB2-amplified breast cancer. Importantly, this candidate emerged exclusively from the proteomic data and was not apparent from genomic profiling. In the present study, this observation should be considered hypothesis-generating. Orthogonal validation would be required to establish a causal role for FAK1 in therapeutic resistance. This example therefore illustrates how proteomic profiling of AllPrep-derived material can generate complementary biological hypotheses when genomic profiling does not reveal an obvious new resistance-associated alteration.

**Fig. 5:**
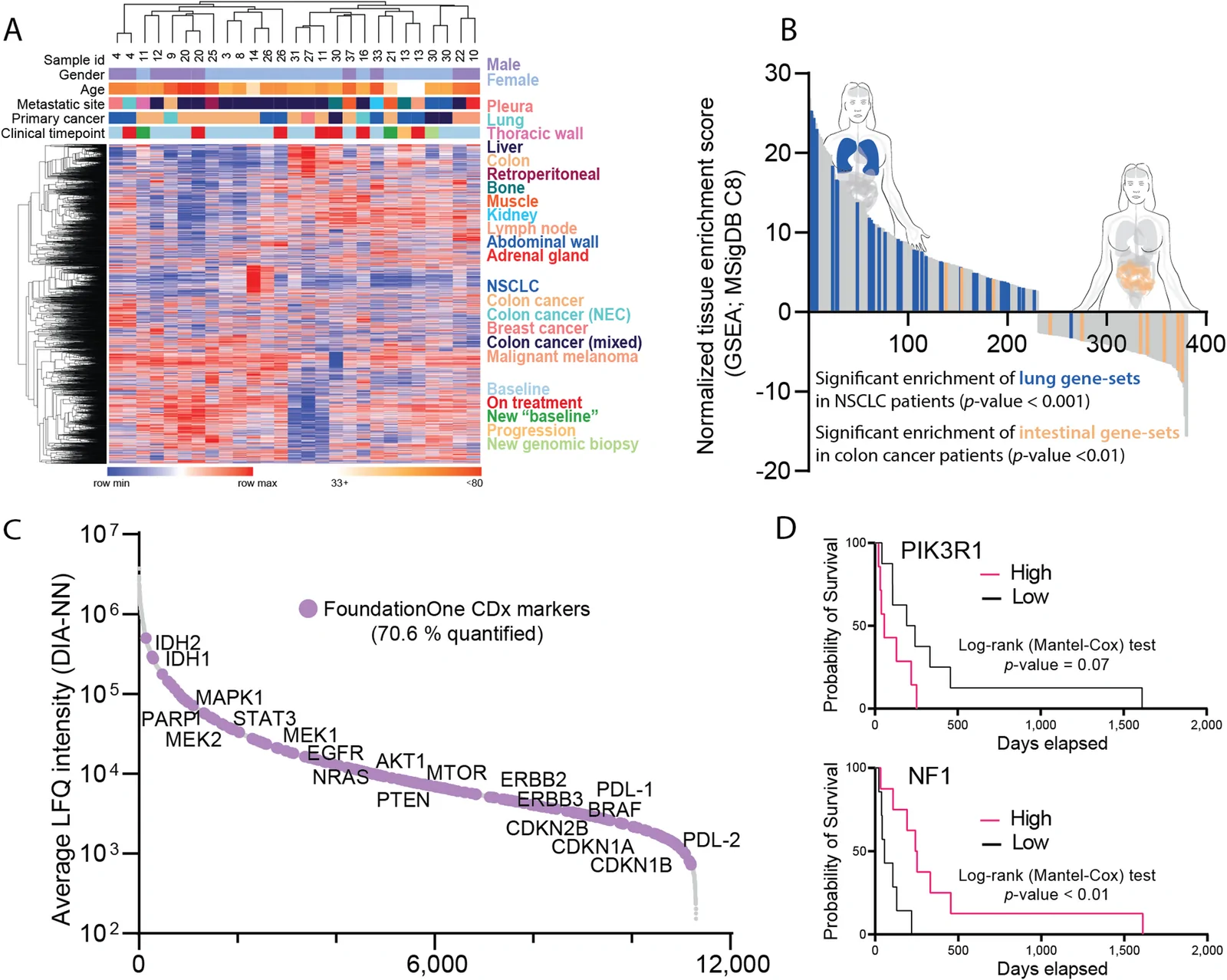
Biological and clinically relevant information retained in AllPrep-derived proteomes. **(A)** Unsupervised clustering of the BRAF V600E–mutated tumor cohort (4,688 protein groups), showing grouping according to patient identity and tumor type, with serial biopsies from the same patients clustering together. **(B)** Gene set enrichment analysis showing tissue-associated signatures, including intestinal signatures in colorectal cancers and lung-associated signatures in non–small cell lung cancer samples. **(C)** Comparison with the FoundationOne^®^ CDx gene panel, showing detection of protein products corresponding to 222 of 315 panel genes (~ 71% protein-level coverage). This comparison reflects protein-level representation and not detection of genomic alterations. **(D)** Exploratory survival analysis of samples collected at the study-defined baseline, showing associations between protein abundance and patient outcome. Higher PIK3R1 abundance showed a trend toward poorer outcome (*p* = 0.07), whereas higher NF1 abundance was associated with improved survival (*p* < 0.01). Survival analyses are exploratory and should not be interpreted as validated prognostic biomarkers

We next asked whether these observations extended to the larger BRAF V600E–mutated tumor cohort. Unsupervised clustering grouped samples according to both tumor type and patient identity, while serial biopsies from the same patients clustered together (Fig. 5A), demonstrating preservation of patient- and tumor-associated proteomic profiles. Gene set enrichment analysis further showed expected tissue-associated biology, with colon cancers enriched for intestinal gene sets and non–small cell lung cancers enriched for lung-associated signatures (Fig. 5B).

To assess the representation of clinically relevant cancer proteins more broadly, we compared the proteomic dataset with the FoundationOne^®^ CDx comprehensive genomic profiling panel, which interrogates more than 300 cancer-related genes used in precision oncology [41]. Protein products corresponding to 222 of the 315 genes represented on the panel were quantified, corresponding to approximately 71% protein-level coverage (Fig. 5C). These included numerous established therapeutic targets and cancer-associated proteins. This comparison reflects protein-level representation of the panel gene set and does not assess the sensitivity or specificity of proteomics for detecting genomic alterations.

Finally, exploratory survival analyses performed on samples collected at the study-defined baseline identified potential associations between protein abundance and patient outcomes. Higher NF1 abundance was associated with improved survival (*p* < 0.01), whereas higher PIK3R1 abundance showed a trend toward poorer outcome (*p* = 0.07) (Fig. 5D). Here, “baseline” refers to the baseline time point within the clinical study and should not be interpreted as treatment-naïve disease. Given the limited cohort size and the advanced, previously treated patient population, these findings should be considered hypothesis-generating rather than validated prognostic biomarkers.

Together, these analyses demonstrate that AllPrep-derived proteomes retain patient-, tumor-, and tissue-associated biological structure while providing access to proteins of potential clinical relevance. These findings support the use of residual AllPrep material for biologically informative and translational proteomic studies.

## Discussion

Comprehensive molecular characterization of tumors increasingly requires the integration of multiple molecular layers derived from the same biological specimen. In heterogeneous tumors, spatial differences within a lesion can result in substantial variability between adjacent biopsy fragments. When genomic, transcriptomic, and proteomic analyses are performed on different pieces of tissue, correlations between these molecular layers may therefore be difficult to interpret.

The clinical cohort consisted of patients with advanced malignancies who had exhausted standard treatment options and was heterogeneous with respect to prior treatment; consequently, exploratory biological and outcome associations should not be interpreted as representing treatment-naïve tumor biology.

Although integrated workflows for DNA and RNA extraction from the same biopsy are well established, obtaining high-quality proteomic information from the identical material remains challenging. Existing approaches often rely on serial sectioning of FFPE tissue or specialized microscale workflows that allow simultaneous proteogenomic measurements [42]. While effective, these methods can be technically demanding and may require specialized equipment or complex sample handling procedures.

The workflow presented here addresses this challenge by utilizing the protein-rich flowthrough generated during routine Qiagen AllPrep DNA/RNA extraction. Because this fraction is produced during the same lysis step used for nucleic acid purification, it provides a direct opportunity to obtain proteomic information from exactly the same biopsy specimen used for genomic and transcriptomic profiling. Importantly, this protein fraction is typically discarded in standard workflows, meaning that proteomic analyses can be performed without requiring additional biopsy material.

Our results demonstrate that with optimized precipitation and cleanup procedures, these fractions yield high-quality proteomes suitable for deep mass spectrometry–based analysis. Although the Advanced Protein Purification buffer supplied with the Qiagen kit proved suboptimal for downstream proteomics applications, acetone precipitation combined with optimized solubilization and digestion conditions enabled efficient recovery of proteins from the flowthrough fractions. The guanidine-based lysis buffer used in the AllPrep protocol appears to preserve protein integrity effectively, likely by rapidly denaturing proteases and preventing post-collection degradation.

A direct paired comparison between AllPrep-derived protein fractions and a conventional dedicated proteomic lysis procedure was not performed in this study. The available clinical biopsies had been processed prospectively for genomic and transcriptomic profiling, and paired biopsy material processed specifically for proteomics was therefore not available. Accordingly, our data establish the feasibility, analytical depth, stability, and reproducibility of proteomic analysis from the residual AllPrep fraction, but do not establish quantitative equivalence to conventional protein extraction workflows.

An important advantage of the workflow is its compatibility with long-term storage. We observed no evidence of protein degradation or increased oxidative modifications in samples stored at − 80 °C for up to five years. This finding suggests that existing biobanks containing stored AllPrep protein fractions represent valuable resources for retrospective proteomic studies.

In addition to demonstrating the feasibility of proteomic analysis from these samples, our study highlights the potential clinical relevance of the resulting datasets. The proteomes quantified numerous cancer-associated proteins, including protein products corresponding to more than 70% of the genes represented on the FoundationOne^®^ CDx panel. This comparison reflects protein-level representation of the panel gene set and should not be interpreted as sensitivity or specificity for detecting genomic alterations. Furthermore, phosphoproteomic analyses revealed thousands of phosphorylation events associated with key oncogenic signaling pathways. Because phosphorylation often reflects pathway activity more directly than gene expression levels, this additional molecular layer may provide important insights into tumor biology and therapeutic response.

Another key strength of the workflow is its scalability. The compatibility of the AllPrep-derived protein fractions with automated bead-based protein aggregation capture (PAC) enabled high-throughput sample preparation on the KingFisher Flex platform. Using automated sample preparation and short-gradient liquid chromatography methods, we demonstrated that deep proteomic coverage can be achieved while maintaining high analytical throughput. Implementation of the Orbitrap Astral platform combined with Evosep chromatography enabled quantification of more than 10,000 proteins per sample at a throughput of up to 60 samples per day. Such throughput makes it feasible to apply this workflow to large clinical cohorts, which is essential for biomarker discovery and translational proteomics research.

Previous studies have explored the feasibility of performing proteomic analyses on protein fractions remaining after nucleic acid extraction, including workflows based on the Qiagen AllPrep system [43–47]. In parallel, several multi-omics protocols have been proposed to enable simultaneous isolation of DNA, RNA, and proteins from the same specimen [48–50]. However, many of these approaches rely on specialized labeling, additional fractionation steps, or workflows that are not routinely implemented in clinical molecular diagnostics laboratories. In contrast, the workflow described here builds directly on the widely used Qiagen AllPrep extraction procedure and leverages the protein-rich flowthrough generated during routine DNA/RNA purification. Importantly, the samples analyzed in this study originate from a clinical precision oncology program in which biopsies undergo genomic and transcriptomic profiling for molecular tumor board evaluation. As a result, the proteomic and phosphoproteomic analyses presented here originate from an existing clinical precision-oncology workflow and could potentially be integrated with genomic data to support multi-omic interpretation in tumor board discussions.

It should be noted that phosphoproteomic depth was dependent on peptide input, with the greatest depth observed at approximately 75–100 µg in the present dataset. However, phosphopeptides were successfully quantified from inputs as low as 29 µg, indicating that lower-input samples remain analytically feasible, albeit at reduced depth. Further optimization of low-input enrichment strategies may improve applicability to very small clinical biopsies.

A key practical advantage of this approach is that proteomic and phosphoproteomic measurements can be generated from the same biopsy material already used for genomic and transcriptomic profiling, without requiring a separate biopsy fragment dedicated to proteomics. This creates opportunities for integrated translational multi-omic studies and retrospective analysis of archived AllPrep flowthroughs. The present study establishes the analytical feasibility of this approach; whether addition of proteomic information improves treatment selection or Molecular Tumor Board decision-making will require dedicated prospective clinical evaluation.

In summary, we demonstrate that the residual protein fraction generated during Qiagen AllPrep DNA/RNA extraction can serve as a robust and scalable source of material for deep proteomic and phosphoproteomic analysis. Using this workflow, we achieved quantification of more than 10,000 proteins and thousands of phosphorylation sites from clinical tumor biopsies, even after long-term storage. The approach represents another step toward integrated multi-omic profiling from a single biopsy specimen and provides a practical resource for translational and clinical proteogenomic research.

## Materials and methods

### Biopsy collection

The study population consisted of patients with advanced solid tumors who had exhausted standard treatment options and were referred to the Phase I Unit at Copenhagen University Hospital, Rigshospitalet (Denmark). Fresh tumor tissue was obtained from metastatic lesions by 18-gauge core needle biopsy or surgical resection under local anesthesia.

For each lesion, three samples were typically collected. Two biopsies were preserved in RNAlater for nucleic acid extraction, while one sample was formalin-fixed and paraffin-embedded for histopathological evaluation. The study was conducted in accordance with the Declaration of Helsinki. For “Ethics Approval and Consent to Participate” please see the “Declarations” section below.

### Recovery of protein fractions

Protein fractions were obtained as residual flowthroughs remaining after sequential DNA and RNA extraction using the Qiagen AllPrep DNA/RNA extraction kit (catalog number 80004). DNA and RNA extractions were performed at the Department of Genomic Medicine at Copenhagen University Hospital. Each fraction contained approximately 800 µL total volume, out of which 250 µL was allotted for subsequent peptide extraction.

Since 2013, the resulting protein-rich flowthrough fractions have been routinely preserved at − 80 °C for potential downstream analyses. These fractions contain guanidine salts, reducing agents such as dithiothreitol (DTT), and variable amounts of ethanol depending on minor procedural variations during extraction.

Protein precipitation was performed using either the Advanced Protein Purification (APP) buffer supplied with the Qiagen kit or acetone precipitation. For APP precipitation, equal volumes of sample and APP buffer were mixed and incubated at room temperature for 10 min before centrifugation at 20,000 × g for 10 min. Pellets were washed with 70% ethanol and briefly air-dried.

For acetone precipitation, four volumes of cold acetone were added to each sample, followed by overnight incubation at − 20 °C. Samples were centrifuged at 20,000 × g for 10 min at 4 °C, washed with acetone, and air-dried prior to resuspension.

### Protein solubilization and digestion

Protein pellets were resuspended in buffers including 1% sodium deoxycholate (SDC), 1–4% SDS, 50 mM Tris-HCl (pH 8.0), or 8 M urea. Resuspension was assisted by vigorous pipetting and sonication using a Bioruptor Plus instrument for 15 cycles of 30 s on and 30 s off.

Proteins were reduced with dithiothreitol and alkylated with chloroacetamide prior to enzymatic digestion. Sequential digestion was performed using Lys-C and trypsin, followed by overnight incubation with agitation.

Alternative workflows using the Protifi S-Trap digestion system were also evaluated according to the manufacturer’s instructions, excluding SDS-containing buffers due to incompatibility with guanidine-containing samples.

Protein aggregation capture workflows were performed using a KingFisher Flex system with MagReSyn Hydroxyl beads [51]. Proteins were aggregated in the presence of acetonitrile and digested using Lys-C and trypsin before peptide recovery. This automated PAC workflow was used for the high-throughput Orbitrap Astral experiment.

### Peptide cleanup and quantification

Peptides were purified using either SDB-RPS StageTips or Sep-Pak C18 cartridges [16]. After washing with trifluoroacetic acid and isopropanol-containing buffers, peptides were eluted using acetonitrile-containing solutions, vacuum-dried, and reconstituted in 3% acetonitrile with 0.1% formic acid for LC–MS analysis.

Protein concentrations were measured using the Pierce BCA protein assay kit. Peptide purity and concentration were assessed using NanoDrop spectrophotometry.

### Phosphopeptide enrichment

Phosphopeptide enrichment was performed using immobilized metal affinity chromatography (IMAC) with Fe(III)-NTA cartridges on the Agilent AssayMAP Bravo platform [52]. Peptides were loaded in 80% acetonitrile with 0.1% trifluoroacetic acid and processed using the automated phosphopeptide enrichment workflow.

Enriched phosphopeptides were eluted in acidic buffers and stored at 4 °C prior to LC–MS analysis.

### Liquid chromatography and mass spectrometry

Peptides were separated using either an EASY-nLC 1200 system or an Evosep One LC platform. EASY-nLC analyses employed in-house packed 50 cm C18 columns with gradient lengths ranging from 70 to 145 min. Evosep analyses used disposable Evotip traps with standardized gradients corresponding to 30 or 60 samples per day.

Proteomic measurements were performed using Orbitrap Exploris 480, Bruker timsTOF Pro, timsTOF SCP, or Orbitrap Astral mass spectrometers depending on the experiment. Data-dependent acquisition methods were used for initial feasibility analyses, while data-independent acquisition workflows including diaPASEF were implemented for large-scale quantitative analyses [19–21].

Peptide loading amounts were optimized independently for each LC–MS configuration to maximize analytical performance under the respective chromatographic and acquisition conditions. Both the final timsTOF DIA and Orbitrap Astral workflows used 100 ng peptide input.

Accordingly, cross-platform comparisons in this study are intended to illustrate achievable performance under optimized setup-specific conditions rather than constitute controlled instrument benchmarking using identical peptide inputs.

### Data processing and bioinformatics

DDA raw files were processed in MaxQuant v1.6.7 using the Andromeda search engine [22, 53] and the UniProt human proteome (UP000005640_9606) as reference. Label-free quantification was performed using MaxLFQ [54], with FDR < 1%.

DIA data were analyzed with DIA-NN v1.8.0.1 [23] for proteomes and Spectronaut v14 (Biognosys) for phosphoproteomes. DIA-NN analyses used 15 ppm tolerances and tryptic specificity, with q-value filtering < 1% [55, 56]. Spectronaut applied the “BGS Phospho PTM Workflow” with 1% FDR thresholds and PTM localization ≥ 0.75. Proteome and phosphoproteome abundance measurements were analyzed as separate molecular layers and were not directly normalized against one another. Astral DIA data were processed using Spectronaut v19 [57] under default directDIA settings.

All searches used UniProt FASTA databases (UP000005640_9606.fasta and UP000005640_9606_additional.fasta), incorporating canonical and alternative isoform sequences [58].

### Statistical analysis

Proteomic datasets were log2-transformed and normalized using median absolute deviation–based approaches. Hierarchical clustering and heatmap visualization were performed using the Morpheus software platform [59].

Open modification searches were conducted using pFind [60], and peptide quantification was performed using pQuant [61]. Clinical and proteomic data integration was performed using the Clinical Knowledge Graph framework [62], with visualization in Cytoscape [63]. FAK1 has been associated with trastuzumab resistance in ERBB2-amplified breast cancer [64].

Kaplan–Meier survival analyses were conducted using GraphPad Prism. Gene set enrichment analyses were performed using the single-sample GSEA method with MSigDB cell-type signatures [65].

## Supplementary Information

Supplementary Material 1.

Supplementary Material 2.

## Data Availability

Proteomics datasets generated in this study have been deposited in the PRIDE repository [66] under accession numbers PXD041313 and PXD056337. These datasets include the primary AllPrep proteomic analyses as well as additional phosphoproteomic and Astral platform datasets associated with related CoPPO studies [67].

## Declarations

### Prior Presentation

Earlier versions of this study were previously available on the bioRxiv preprint server: https://www.biorxiv.org/content/https://doi.org/10.1101/2023.05.12.540582v2.
