# Supplementary material for: Scalable clinical proteomics from DNA/RNA extraction flowthroughs enables integrated proteogenomic profiling from a single cancer biopsy
Source: Clin Proteomics. 2026 Sep 20;23:45. doi: 10.1186/s12014-026-09632-1 (PMC13616122; doi:10.1186/s12014-026-09632-1)
Supplement: Supplementary file 1 [file 12014_2026_9632_MOESM2_ESM.docx]

**Scalable Clinical Proteomics from DNA/RNA Extraction Flowthroughs Enables Integrated Proteogenomic Profiling from a Single Cancer Biopsy**

**Authors:** Filip Mundt Madsen, Annelaura Bach Nielsen, Juanjuan Wang, Josephine Kerzel Duel, Christina Westmose Yde, Martina Amnitzbøll Eriksen, Ulrik Lassen, Finn Cilius Nielsen, Kristoffer Rohrberg, Matthias Mann

# **Abstract**

**Background:**
Precision oncology has largely relied on genomic and transcriptomic sequencing to guide therapy selection in patients with advanced cancer. However, these molecular layers do not fully capture the functional state of tumors, as proteins represent the active effectors of cellular processes and signaling pathways. Integrating proteomic and phosphoproteomic measurements with genomic data has therefore emerged as an important objective in translational oncology. In routine clinical workflows, tumor biopsies are frequently processed using DNA/RNA extraction kits such as the Qiagen AllPrep system, which generates a protein-rich flowthrough that is typically discarded.

**Methods:**
Here we systematically evaluated whether the residual protein fraction generated during Qiagen AllPrep DNA/RNA extraction can serve as a reliable source for mass spectrometry–based proteomics in clinical tumor biopsies. Within the Copenhagen Prospective Personalized Oncology (CoPPO) precision oncology trial, residual protein fractions from metastatic cancer biopsies were recovered, precipitated, and processed using optimized workflows for liquid chromatography–mass spectrometry. We established procedures for protein recovery, digestion, and peptide cleanup, and evaluated multiple acquisition strategies including data-dependent acquisition (DDA) and data-independent acquisition (DIA). We further assessed sample stability during long-term storage, analytical reproducibility, scalability, and compatibility with phosphoproteomics.

**Results:**
Optimized single-shot workflows consistently generated deep proteomes from minimal biopsy material, quantifying more than 10,000 protein groups in DIA analyses. Protein integrity was preserved in samples stored at −80 °C for up to five years. The resulting proteomes captured tissue-specific biological signatures and quantified 71% of clinically actionable cancer genes included in the FoundationOne® CDx panel. Phosphoproteomic analyses identified more than 10,000 phosphorylation sites associated with key oncogenic signaling pathways including AKT1, BRAF, and EGFR. Implementation of short liquid chromatography gradients enabled high-throughput analysis of up to 60 proteomes per day without compromising data quality.

**Conclusions:**
Residual protein fractions from Qiagen AllPrep DNA/RNA extraction provide a robust and scalable resource for deep proteomic and phosphoproteomic profiling from the same biopsy used for genomic and transcriptomic analyses. This workflow enables integrated proteogenomic characterization from a single clinical sample and provides a practical route toward incorporating proteomics into precision oncology and Molecular Tumor Board decision-making.

# **Supplementary Figures**

**Supplementary Fig. S1. Proteome depth and global proteomic structure of AllPrep-derived tumor samples.**

(**A**) Numbers of quantified peptides and protein groups obtained from 27 tumor samples harboring BRAF mutations. Samples had been stored at −80 °C for periods ranging from one to five years prior to proteomic analysis. Comparable numbers of identified peptides and proteins across storage times indicate that long-term storage does not substantially affect proteome recovery from AllPrep-derived protein fractions. (**B**) Principal component analysis (PCA) of the 27 tumor proteomes showing clustering according to primary tumor type and metastatic site. The analysis demonstrates that proteomic profiles derived from AllPrep flowthrough fractions retain biologically meaningful structure and are suitable for downstream clinical proteomics analyses. PC = principal component.

**Supplementary Figure S2. Concordance between proteome and phosphoproteome measurements in AllPrep-derived tumor samples.**

Unsupervised hierarchical clustering of the 27 BRAF-mutated tumor samples based on either total proteome measurements (**left**) or phosphoproteome measurements following IMAC enrichment (**right**). Clustering was performed using one-minus Pearson correlation. The color coding of dendrogram clusters is matched between the two heatmaps, indicating that the same patient samples cluster together in both proteomic and phosphoproteomic datasets. For example, the yellow cluster located at the left side of both dendrograms represents the same group of patient samples in both analyses. These results demonstrate that AllPrep-derived samples produce consistent biological structure across both proteomic and phosphoproteomic layers, supporting the robustness of the workflow for integrated clinical proteomics analyses.
